# AI-assisted automatic MRI-based tongue volume evaluation in motor neuron disease (MND)

**DOI:** 10.1007/s11548-024-03099-x

**Published:** 2024-03-27

**Authors:** Ina Vernikouskaya, Hans-Peter Müller, Albert C. Ludolph, Jan Kassubek, Volker Rasche

**Affiliations:** 1 Department of Internal Medicine II, Ulm University Medical Center, Albert-Einstein-Allee 23, 89081 Ulm, Germany; 2 Department of Neurology, University of Ulm, Ulm, Germany; 3 German Center for Neurodegenerative Diseases (DZNE), Ulm, Germany; 4 Core Facility Small Animal MRI, University of Ulm, Ulm, Germany

**Keywords:** Tongue segmentation, Triplanar U-Net, Consensus model, MRI, Motor neuron disease (MND)

## Abstract

**Purpose:**

Motor neuron disease (MND) causes damage to the upper and lower motor neurons including the motor cranial nerves, the latter resulting in bulbar involvement with atrophy of the tongue muscle. To measure tongue atrophy, an operator independent automatic segmentation of the tongue is crucial. The aim of this study was to apply convolutional neural network (CNN) to MRI data in order to determine the volume of the tongue.

**Methods:**

A single triplanar CNN of U-Net architecture trained on axial, coronal, and sagittal planes was used for the segmentation of the tongue in MRI scans of the head. The 3D volumes were processed slice-wise across the three orientations and the predictions were merged using different voting strategies. This approach was developed using MRI datasets from 20 patients with ‘classical’ spinal amyotrophic lateral sclerosis (ALS) and 20 healthy controls and, in a pilot study, applied to the tongue volume quantification to 19 controls and 19 ALS patients with the variant progressive bulbar palsy (PBP).

**Results:**

Consensus models with softmax averaging and majority voting achieved highest segmentation accuracy and outperformed predictions on single orientations and consensus models with union and unanimous voting. At the group level, reduction in tongue volume was not observed in classical spinal ALS, but was significant in the PBP group, as compared to controls.

**Conclusion:**

Utilizing single U-Net trained on three orthogonal orientations with consequent merging of respective orientations in an optimized consensus model reduces the number of erroneous detections and improves the segmentation of the tongue. The CNN-based automatic segmentation allows for accurate quantification of the tongue volumes in all subjects. The application to the ALS variant PBP showed significant reduction of the tongue volume in these patients and opens the way for unbiased future longitudinal studies in diseases affecting tongue volume.

## Introduction

Amyotrophic lateral sclerosis (ALS), the most common adult motor neuron disease (MND), is characterized by a progressive loss of motor neurons that leads to progressive pareses, respiratory failure, and death mostly within 3 to 5 years after its onset [1]. Bulbar dysfunction, characterized by tongue wasting and fasciculation, accompanied by flaccid dysarthria and dysphagia, is emerging in the vast majority of patients during the advanced phases of the disease [1, 2], as one major factor that determines a patient’s prognosis [3]. Prognostic biomarkers of ALS are needed, particularly of bulbar involvement, which is one of the key determinants of long-term prognosis and survival in this disorder [4]. Progressive bulbar palsy (PBP) is an ALS variant in which patients show an isolated bulbar onset with a progressive affection of the lower cranial nerves including tongue atrophy [5] before they develop spinal symptoms of MND.

Quantitative noninvasive imaging-based assessment of the severity of tongue volume loss requires conduction of longitudinal studies measuring several tongues features with specialized instruments including magnetic resonance imaging (MRI) or high-resolution ultrasound. Several case reports and few systematic imaging studies have suggested structural tongue measures in the course of ALS [3, 6, 7]. Specifically, T1 sequences were used to assess atrophy, fibrosis and fatty degeneration, and a previous large-scale study suggested that in vivo sonography and region-of-interest (ROI)-based MRI tongue measures could aid as biomarkers to reflect bulbar and motor function impairment in ALS [7]. Although it has been previously shown by ultrasound that the tongue thickness in a group of 18 ALS was lower than that of healthy controls [3], tongue size and shape can significantly vary across subjects and longitudinal studies need to be performed to investigate the tongue muscle atrophy in diseases like ALS.

In order to access measurements like volume, thickness, or shape of a given structure, the anatomy needs to be segmented, which is a time-consuming and error-prone process when performed manually. To reduce the time and subjectivity of medical segmentations and consequently improve reliability, automatic segmentation methods based on deep convolutional neural networks (CNNs) were used [8]. It has been shown by the authors of the nnU-Net, i.e., a framework relying on 2D and 3D U-Nets that automatically configure themselves [9] that plain end-to-end CNNs with U-Net like architectures perform exceptionally well in most biomedical image segmentation tasks.

After several studies based on CNNs for MRI and ultrasound images of the vocal tract for understanding speech production [10–14], more studies on using semantic segmentation based on deep CNNs for tongue segmentation have been recently conducted, and the effect is better than most of the traditional image segmentation methods [15, 16]. However, there are still limitations in those methods, e.g., including image preprocessing such as image enhancement [17] making the whole segmentation process more complex or brightness discrimination [18] reducing the ability of generalization as a deep learning-based model.

The aim of the present study was to adapt the CNN model of U-Net architecture to MRI data of the tongue (which are included in routinely acquired volume-rendering scans of the human head) with the final goal to obtain an automated pipeline for determination of tongue atrophy in neurologic diseases. In our approach, the T1-weighted MRI images from the 3D volumes are processed slice-wise across the axial, sagittal, and coronal planes with the CNN of U-Net like architecture, and the predictions from the three orthogonal orientations are merged using different voting strategies integrating more 3D information into the 2D model. Compared to the original triplanar U-Net approach where three orientation-specific U-Nets are trained [19], we utilize a single U-Net which is trained on axial, sagittal, and coronal slices [20], allowing to share common features across orientations. Furthermore, we investigate the sensitivity of different voting strategies for merging the predictions from different orientations. We developed our approach using 40 datasets available with reference segmentation of the tongue (20 from healthy controls and 20 from MND patients diagnosed with ALS) and applied it in a group comparison study comprising further 19 controls and 19 MND patients diagnosed with PBP.

## Methods

### MRI dataset

**Fig. 1:**
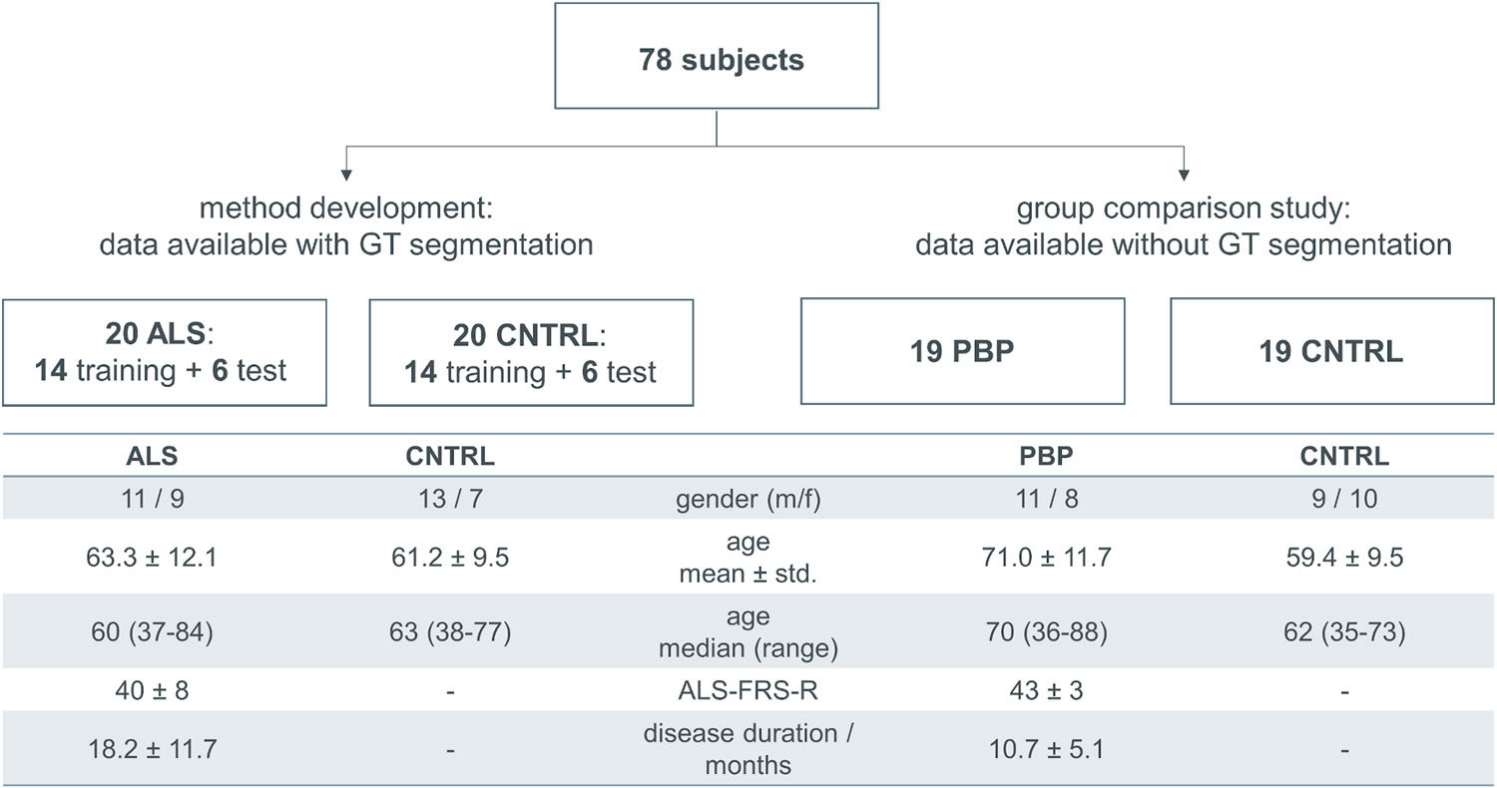
Overview of all available datasets subdivided into data used for method development and method application in a group comparison study. Demographic characteristics for all data groups are listed in the respective table. ALS-FRS-R—revised ALS functional rating scale [21], ALS—amyotrophic lateral sclerosis patients, PBP—progressive bulbar palsy patients, CNTRL—controls

Seventy-eight T1-weighted whole head MRI datasets acquired on a 1.5 T MRI scanner (Symphony, Siemens Medical, Erlangen, Germany) with a T1-weighted 3D MPRAGE sequence as a standardized clinical MRI examination protocol for patients with MND were available for this study. Data were obtained from the MRI database of the Department of Neurology, University of Ulm, Germany. The respective ethics application includes the recording and the analysis of MRI data, irrespective of the analysis technique; no additional MRI scans have been performed for the current study. The field-of-view of the T1-weighted images of the head usually also covers the tongue so that these images could be used for segmentation of the tongue. T1-weighted scans that did not cover the tongue were not used in this study. In addition, motion artifacts due to tongue movement could not be excluded although subjects were not instructed to keep the tongue still. Figure 1 provides overview of all available datasets with the corresponding demographic data for each group.

Forty datasets including 20 healthy subjects without any neurologic/psychiatric disease or other medical condition and 20 patients with sporadic ALS who were diagnosed with definite, probable, or possible ALS according to revised E1 Escorial criteria [22] recruited in the outpatient and inpatient settings of the Department of Neurology, University of Ulm, Germany were available with the corresponding reference segmentation of the tongue. For methodological development, the data samples from controls and ALS patients were randomly split into training and test datasets at a ratio of 70%/30% at subject level, resulting in 14 training and 6 test datasets from each group.

Further, 19 patients diagnosed with PBP, who met the diagnostic criteria for PBP, and 19 controls were investigated in the group comparison study. The MRI data were part of a previous study with a different research focus [23]. All PBP patients showed an isolated bulbar onset with a progressive affection of the lower cranial nerves causing dysarthria and/or dysphagia, tongue wasting and fasciculation before they developed spinal MND symptoms. To be eligible, subjects had to fulfill the following criteria: no family history of MND, no clinical diagnosis of frontotemporal dementia, no other major systemic, psychiatric or neurologic illnesses, no history of substance abuse. Further mandatory criteria for inclusion were negative tests for other neuromuscular diseases and for infections of the central nervous system, and routine MRI scans excluded any brain abnormalities indicating a different etiology of the clinical symptoms. These data were available without the corresponding ground truth labels.

### Preprocessing and generation of ground truth segmentations

**Fig. 2:**
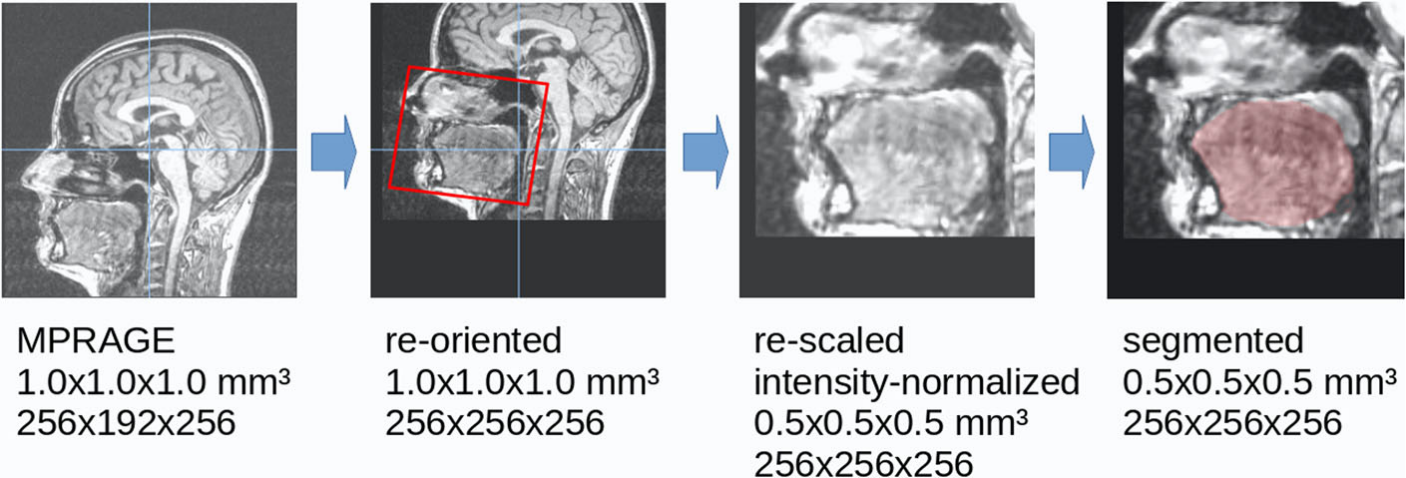
Preprocessing pipeline. The original MPRAGE images were (if necessary) rescaled to 1.0 × 1.0 × 1.0 mm^3^ and reoriented with the tongue in the center of the dataset. After intensity normalization segmentation was performed in a 256 × 256 × 256 matrix (resolution 0.5 × 0.5 × 0.5 mm^3^). The central sagittal slices are displayed

For image preprocessing and creating a ground truth label the software package *Tensor Imaging and Fiber Tracking (TIFT)* was used, expanded by a volumetric extension package [24]. In the preprocessing pipeline, original 3D MPRAGE volumes were first rescaled to a 256 × 256 × 256 matrix with an isotropic resolution of 1.0 × 1.0 × 1.0 mm^3^. After rescaling, the data were reoriented with the palatal tip in the center of the matrix and with the nose pointing to the right in the sagittal image. Data were intensity-normalized with z-score normalization based on the mean and standard deviation of the means of all subjects who participated in the study. The mean intensity value for the individual subjects was determined in an area defined by a matrix of 128 × 128 × 128 voxels with the tongue as the center (note: this area was the same for all subjects). A visualization is provided in Fig. 2. The segmentation was then carried out in a rescaled matrix of 256 × 256 × 256 voxels with a resolution of 0.5 × 0.5 × 0.5 mm^3^. The segmentation of the ground truth data of the tongue was performed manually using a 3D intensity threshold-based marking tool. Data were displayed in parallel in axial, sagittal, and coronal views and the tongue was manually marked (Fig. 2, right) by a 3D painting/drawing tool implemented in the *TIFT* software platform by a trained operator (HPM) and controlled by a medical expert (JK). The number of slices covering the tongue varied between subjects and orientations with an average number of 120 slices in axial, 95 slices in sagittal, and 143 slices in coronal orientation.

### Model training

**Fig. 3:**
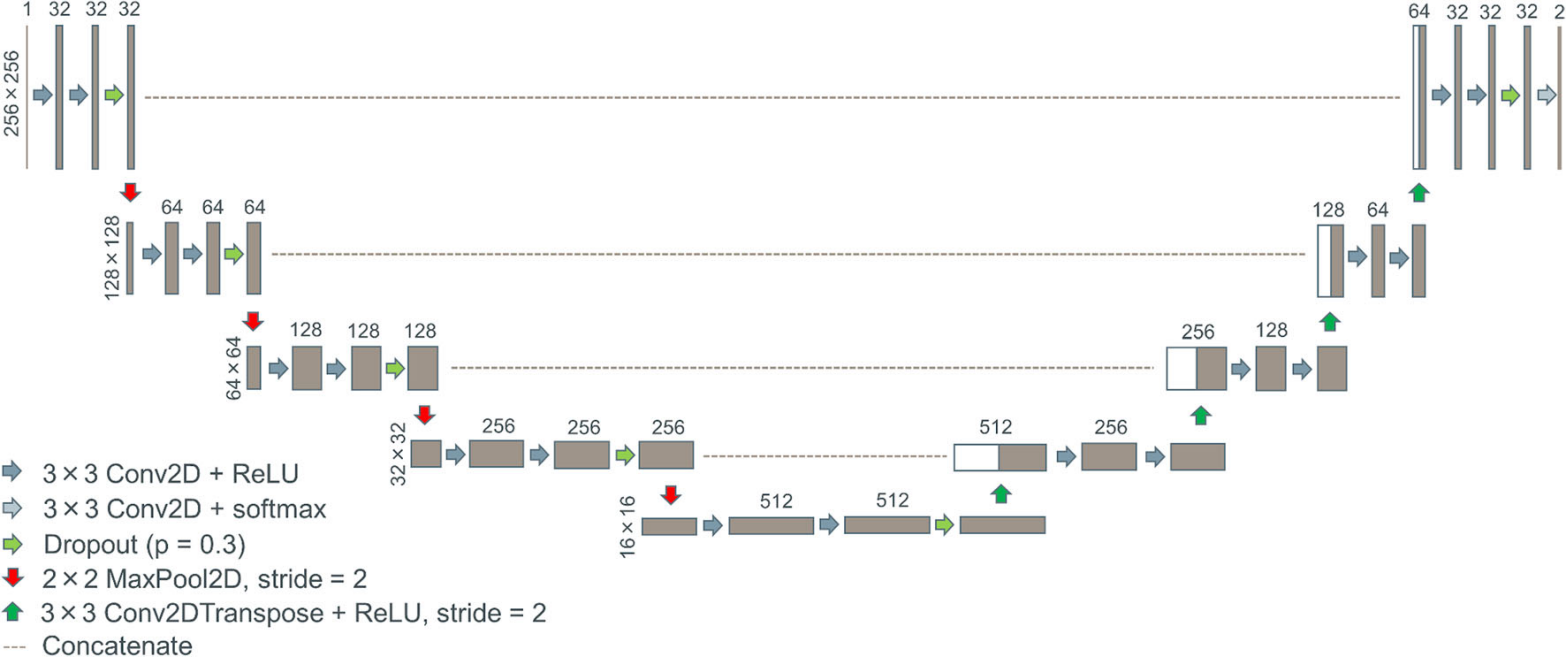
Modified U-Net architecture. Each brown box corresponds to a multi-channel feature map with the number of channels denoted on top of the box and x–y-size provided at the left edge of the box. White boxes represent copied feature maps. Arrows denote different operations with the corresponding settings

For training we used U-Net model implemented from scratch. In contrast to original architecture with five convolutional blocks on each branch, the number of feature channels in the contracting path was reduced to 32, 64, 128, 256, and 512, respectively. To increase the network generalization and reduce overfitting, a dropout layer was applied after repeated 3 × 3 convolutional layers with ReLU activation in each downsampling step. The modified U-Net architecture with the corresponding layers’ settings is shown in Fig. 3.

The training was performed along 50 epochs using early stopping where the training was stopped when the validation loss was observed to have ceased improving for 10 consecutive epochs with a batch size of 16 images per pass. The loss function was based on the categorical cross entropy and Adaptive Moment Estimation (Adam) with the learning rate of 10^–4^ and remaining hyperparameters kept with their default Keras values was used as the optimizer. Mean Intersection over Union (IoU) was used as metric to evaluate the model. Fivefold cross-validation strategy was applied for training, where 20% of available data was hold-out at each fold as validation set.

### Inference

**Fig. 4:**
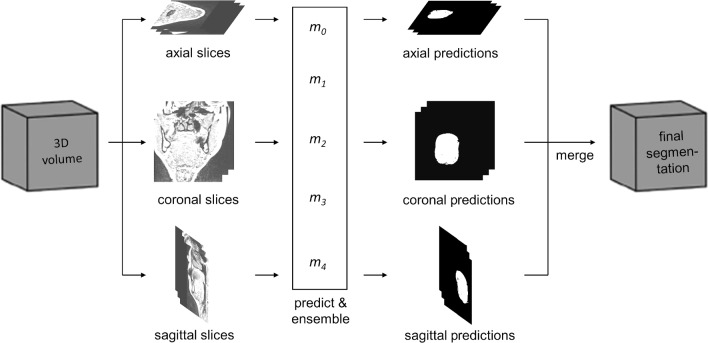
Segmentation pipeline using the triplanar U-Nets *m*_0_, …, *m*_4_ trained on slices from the three orthogonal planes in fivefold cross-validation process. For every individual orientation the predicted probability vectors at each fold are weighted with the corresponding fixed weights and averaged to obtain final softmax score. In the final step, the predictions of the individual orientations are merged to yield the final segmentation

For inference, the weighted ensemble average of all fivefold models with the fixed weights, i.e., the validation IoU at each fold, was used for 2D slice-by-slice segmentation of axial, sagittal, and coronal images, respectively (Fig. 4).

Resulting axial, sagittal, and coronal predictions were merged using four different voting strategies to produce the final segmentation mask. In addition to softmax averaging with equal weights as a baseline approach [19], we compared three different voting strategies in order to find the optimal balance of recall and precision. For these approaches, we first thresholded the softmax scores of each of the three orientations to obtain hard predictions. Then, the following strategies were applied: the exact segmentation of a tongue was defined as the union of the corresponding positive voxels across (a) at least one orientation prediction (union); (b) at least two orientation predictions (majority); (c) all orientation predictions (unanimous voting).

### Evaluation metrics

To evaluate the introduced approaches, performance metrics such as precision, recall, and the principal segmentation metric, i.e., Dice score which is equivalent to F1 score, were calculated via the true positives (TP), false positives (FP), and false negatives (FN). Further, ground truth tongue volumes and tongue volumes predicted by different approaches were compared applying a paired Student’s *t* test according to Shapiro–Wilk test for normality (*p*-value < 0.05 was assumed statistically significant). Finally, we accessed the differences between tongue volumes in the control and the PBP group applying unpaired *t* test or Mann–Whitney U rank test as appropriate depending on the results of Shapiro–Wilk test.

## Results

While having the highest number of TP among all investigated prediction strategies, the most inclusive strategy, i.e., union, achieved the best recall (0.93 on average) due to significantly lower number of FN, but a very low precision (0.78) given by the large number of FP, resulting in an overall Dice score of 0.85. The Dice score for very restrictive, unanimous, voting was similar (0.85) having the highest precision of 0.92 (due to the lowest number of FP), but the lowest recall of 0.80 (due to the highest number of FN). Softmax averaging and majority voting performed best in terms of segmentation accuracy (Dice score of 0.88) due to similarly high precision (0.88) and recall (0.88), outperforming two other merging strategies and improving predictions on single orientations. These results are summarized in Table 1.

**Table 1:** True positives (TP), true negatives (TN), false positives (FP), and false negatives (FN), as well as calculated performance metrics (mean precision, mean recall, and mean F1 score) achieved with predictions on single axial, sagittal, and coronal orientations and after application of consensus models with different merging strategies (softmax averaging, union, majority, and anonymous voting) calculated on 12 test datasets consisting of 6 controls and 6 ALS patients

	Axial	Sagittal	Coronal	Softmax	Union	Majority	Unanimous
TP × 10^5^	6.75 ± 1.20	6.54 ± 1.21	6.54 ± 1.19	6.68 ± 1.22	**7.08 ± 1.28**	6.66 ± 1.21	6.09 ± 1.13
TN × 10^5^	159 ± 1.43	159 ± 1.63	159 ± 1.32	159 ± 1.43	158 ± 1.54	159 ± 1.42	**160 ± 1.40**
FP × 10^4^	13.0 ± 4.11	11.2 ± 6.74	9.29 ± 3.58	8.95 ± 4.66	19.4 ± 5.65	8.99 ± 4.61	**5.09 ± 3.29**
FN × 10^4^	8.87 ± 5.74	11.0 ± 6.40	10.9 ± 6.37	9.58 ± 6.17	**5.53 ± 3.92**	9.77 ± 6.22	15.5 ± 8.04
Precision	0.84 ± 0.05	0.86 ± 0.07	0.87 ± 0.05	0.88 ± 0.06	0.78 ± 0.06	0.88 ± 0.06	**0.92 ± 0.05**
Recall	0.89 ± 0.06	0.86 ± 0.07	0.86 ± 0.07	0.88 ± 0.07	**0.93 ± 0.04**	0.88 ± 0.07	0.80 ± 0.09
F1 score	0.86 ± 0.03	0.86 ± 0.02	0.86 ± 0.03	**0.88 ± 0.03**	0.85 ± 0.03	**0.88 ± 0.03**	0.85 ± 0.04

The best performing method is displayed in “bold”

**Fig. 5:**
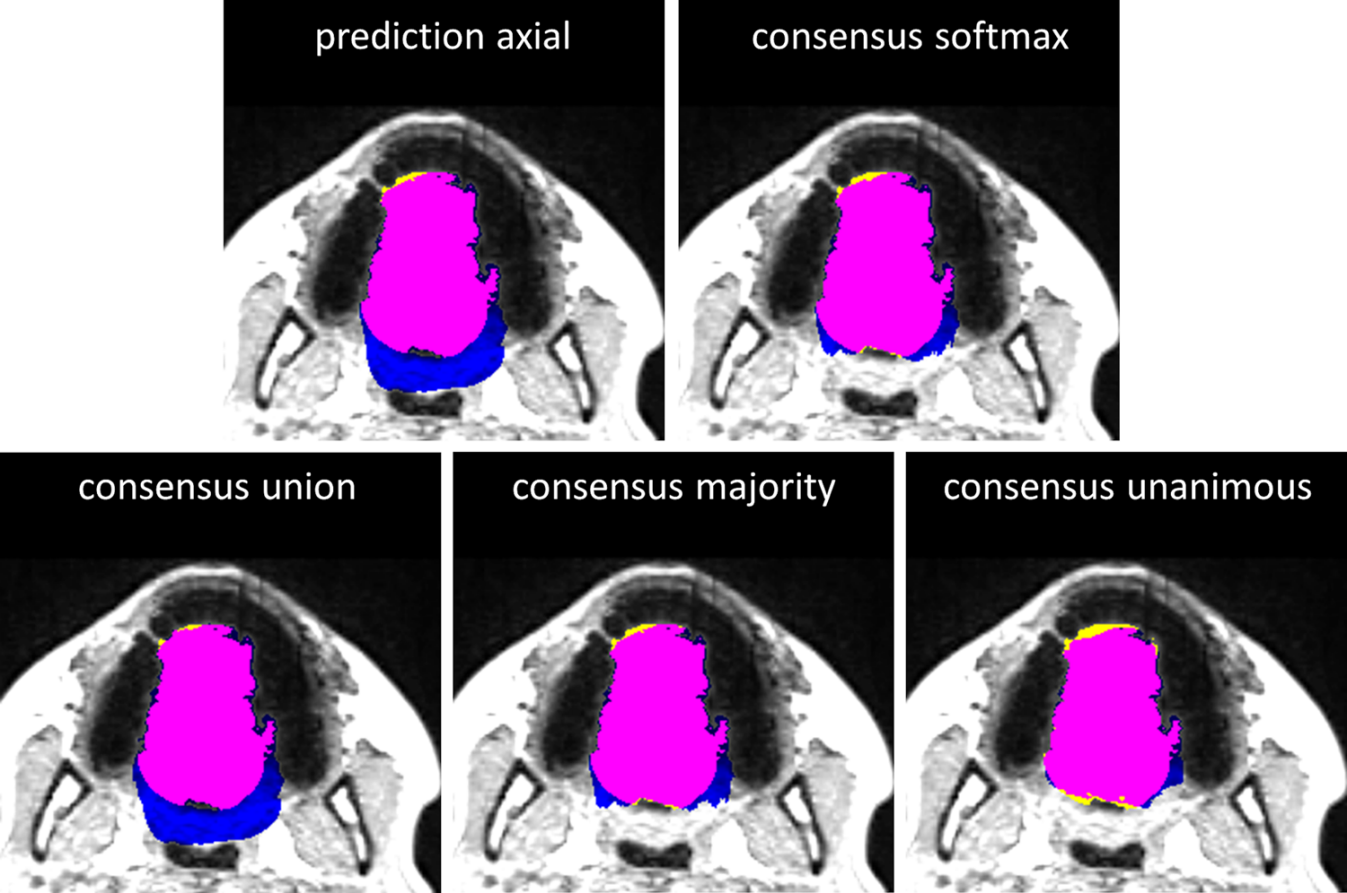
Visual results from a single axial slice of an ALS patient from the test group. Each image represents the overlay of the ground truth tongue mask and predicted mask returned by the axial model as well as consensus models with softmax averaging of three respective orientations, union, majority, and unanimous voting on the respective axial MRI slice. Color coding is as follows: matching pixels between ground truth tongue mask and predicted tongue mask with either approach are shown in magenta, false positives are shown in blue, and false negatives are shown in yellow

Qualitative results from a single MRI slice of ALS patient confirmed results of the confusion matrices and are summarized in Fig. 5. All models provided similar number of true predictions (magenta overlay in Fig. 5). While predicting less false positives (blue overlay) than e.g., the axial model only or the consensus model with union voting and missing very little number of positives (yellow overlay) as compared to e.g., the consensus model with unanimous voting, consensus models with softmax averaging and majority voting performed best.

**Fig. 6:**
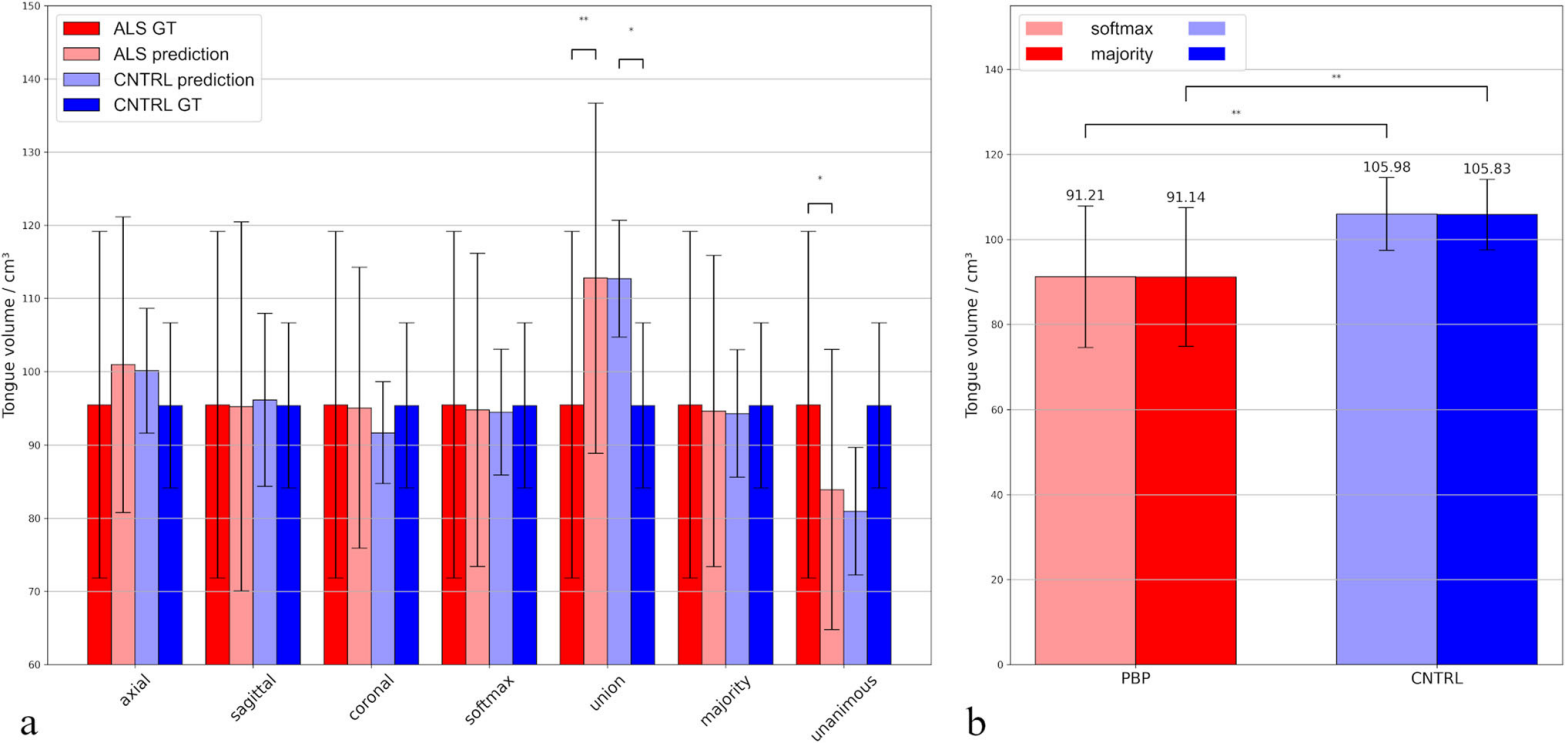
Tongue volume quantification using different prediction strategies. **a** Bar plot comparing average tongue volumes in ALS patients’ (light red bars—prediction, dark red bars—ground truth) and control’s (light blue bars—prediction, dark blue bars—ground truth) groups from the test dataset consisted of 6 subjects each quantified with different prediction strategies. *(*p* < 0.05), **(*p* < 0.005) significance in volume quantification between either approach and ground truth was calculated using paired Student’s t test. **b** Bar plot comparing average tongue volumes of 19 PBP patients and 19 healthy controls quantified with consensus models with softmax averaging (light red and light blue bar, respectively) and majority voting (dark red and dark blue bar, respectively) with black error bars denoting standard deviations. **(*p* < 0.005) significance in volume quantification between both groups with either approach was calculated using unpaired Student’s t test

Differences in volume quantification between each approach and ground truth in the test dataset subdivided into control and ALS subgroups (6 subjects each) are demonstrated in Fig. 6a. Very good accordance was observed between ground truth tongue volumes and predictions in both ALS and controls with consensus models with softmax averaging and majority voting, which obviously outperformed predictions on individual orientations. Highly significant overestimation of tongue volumes in comparison to ground truth in both groups was observed for the consensus model with union voting and underestimation with unanimous voting, especially in the ALS group. The differences in tongue volumes between controls’ and ALS patients’ test groups with either approach were relatively small and statistically not significant.

The analysis at the group level using the best performing consensus models with softmax averaging and majority voting revealed a significant reduction in the quantified tongue volume at *p* = 0.002 in PBP patients (91 ± 16) versus controls (106 ± 8), even in this rather low sized data sample of 19 subjects per group (Fig. 6b).

## Discussion

Accurate delineation of the tongue from low-contrast medical MR images of soft tissue remains a challenge, due to the lack of definitive boundary features separating many of the adjacent soft tissue [25]. Different from the conventional segmentation tasks in nature scene, tongue segmentation is more challenging because of the following issues: (1) large variations of tongue appearance for different patients while higher precision requirement; (2) data imbalance, e.g., small parts of foreground region (tongue body) compared with the background region; and (3) hard sample mining, e.g., lip pixels as the hard samples is hard to be segmented from tongue pixels because the similar appearances and close touch between them [15]. Recent studies demonstrated the applicability of AI methods in tongue segmentation [14, 26]. MRI is a useful modality for the noninvasive quantification of the tongue volume for longitudinal assessments of the muscle atrophy associated with the disease progression e.g., in patients with MND [7] who often present with tongue atrophy as a bulbar sign. Thus, an automatic method providing segmentation accuracy of the tongue comparable to that of an expert can be highly beneficial to reduce manual reading time and efforts.

In this methodological pilot study, we approach this challenge presenting a CNN-based method using single U-Net trained on three orthogonal orientations with consequent merging of respective orientations in a consensus model using different voting strategies. Training of a triplanar network as opposed to training of three orientation-specific networks requires that all slices have identical dimensions which we have ensured by resampling the volume to a regular cube in the preprocessing step. Further, we have observed that single orientation predictions tend to contain many erroneous detections, hence we applied different merging strategies of individual orientations in order to potentially reduce both the numbers of false positives and false negatives and to increase the segmentation accuracy. The most restrictive unanimous merging strategy implying that only pixels that had been confirmed in all three orientations are accepted has been previously suggested to be a key factor for the good performance of lesion segmentation [20]. In our study, unanimous voting strategy achieved the highest precision, i.e., most of the pixels predicted as tongue were true predictions. However, this precision gain was outweighed by the loss in recall showing that this approach missed pixels from the tongue. The opposite approach with union of all orientations achieved very high recall at the contrary, but obviously is limited by the low precision. As a result, both approaches yield significant deviation in segmented tongue volume compared to unbiased ground truth. Softmax averaging of predicted probabilities (which is equivalent to merging with majority voting in case of equal weights for the three respective orientations) performed best, balancing precision and recall better than other models and achieved an average Dice coefficient of 0.88. Very good accordance between ground truth tongue volume and tongue volume provided by consensus models with softmax averaging and major voting was achieved in our test dataset, outperforming all other strategies at the group level.

In our datasets from ALS patients with ‘classical’ spinal manifestation on the one hand and patients with the ALS variant PBP with prominent bulbar syndrome including hypoglossus nerve involvement with consecutive tongue affectation on the other hand, plausible results could be obtained. Using the introduced automatic approach, the assessments in the ‘classical’ spinal ALS showed no significant results versus controls with respect to tongue volumes. These data are in accordance with previous studies, including a study in 206 ALS patients in which the MRI analyses of the tongue for different parameters including sagittal tongue area resulted in only small effect sizes [7]. Obviously, the variability of the tongue involvement as one part of the bulbar syndrome is high in ‘classical’ spinal ALS. In contrast, a highly significant difference in tongue volume was obtained between PBP patients and healthy controls in our study, even though high variability of tongue volumes was also observed at cross-sectional level. In this group with prominent bulbar symptoms, tongue involvement is a major element of the clinical presentation.

## Conclusions

A CNN model of U-Net like architecture was successfully adapted for segmentation of the tongue from routinely acquired MRI scans of the human head. The training on three orthogonal orientations with consequent merging of respective orientations in a consensus model allowed for an automated determination of atrophy of the tongue at the group level. That way, the added value of this study is the future use of the developed pipeline in longitudinal clinical studies for the detection of tongue atrophy in neurologic diseases. To this end, not only larger patient groups have to be investigated, but correlation analyses have to follow with clinical (and perhaps other technical) markers of disease severity and longitudinal progression. More specifically, the structure/volume of the tongue will have to be correlated with tongue function assessments including tongue movement ability and swallowing function.
